# Health work in the context of a pandemic: For a research agenda

**DOI:** 10.1590/0037-8682-0427-2020

**Published:** 2020-11-06

**Authors:** Flávia Regina Souza Ramos, Marcus Vinicius Guimarães Lacerda, Darlisom Sousa Ferreira, Kássia Janara Veras Lima, Igor Castro Tavares, Wagner Ferreira Monteiro, Cleise Maria de Goes Martins, George Lucas Augusto Trindade da Silva, Lucas Lorran Costa de Andrade, Wuelton Marcelo Monteiro

**Affiliations:** 1 Universidade Federal de Santa Catarina, Pós-graduação em Enfermagem, Florianópolis, SC, Brasil.; 2 Universidade do Estado do Amazonas, Programa de Pós-Graduação em Enfermagem em Saúde Pública, Manaus, AM, Brasil.; 3 Universidade do Estado do Amazonas, Programa de Pós-graduação em Medicina Tropical, Manaus, AM, Brasil.; 4 Fundação de Medicina Tropical Dr. Heitor Vieira Dourado, Manaus, AM, Brasil.; 5 Fundação Oswaldo Cruz, Instituto Leônidas e Maria Deane, Manaus, AM, Brasil.; 6 Universidade do Estado do Amazonas, Mestrado Profissional em Enfermagem em Saúde Pública, Manaus, AM, Brasil.; 7 Centro Universitário Luterano de Manaus, Graduação em Enfermagem, Manaus, AM, Brasil.

**Keywords:** Health workers, Pandemic, COVID-19, Health research

## Abstract

**INTRODUCTION:**

The emergence of a pandemic highlights the translational demands regarding science. This communication aims to propose theoretical-methodological elements for research on health work in pandemic context.

**METHODS:**

This reflective essay sets an framework for a research project on health work in Manaus, Amazonas, Brazil.

**RESULTS:**

Three axes or subsidies are presented: the construction of work environments as an analytical component, the approach of ergology as a potential and the centrality of the experiences of the worker-subject.

**CONCLUSIONS:**

New health care challenges require attention to what workers have to say about their forms of confrontation and translation of knowledge.

In recent years, the emergence of new infectious diseases has impacted society and triggered several questions about the assignment of National Public Health Systems to legitimize surveillance and certain healthcare strategies, including the prior identification of new outbreaks and the power to contain them^1^. The circumstances of a sanitary and humanitarian emergency posed by the new coronavirus disease (COVID-19) demonstrate the relevance of multidisciplinary approaches in the construction of measures to cope with COVID-19 and the urgency to integrate epidemiological, clinical, diagnostic, treatment, and prevention analyses for an understanding of the social and cultural construction of the phenomenon and manners of living and working in light of the pandemic.

The urgency and speed of the production and dissemination of information configure the phenomenon called the “infopandemic”-an overabundance of data and knowledge regarding COVID-19. The circulation of information-true and false, largely speculative or inconclusive-occurs at a high speed, usually through unfiltered channels^2^. The threats of this phenomenon demand strategies adopted by the WHO as a way to combat disinformation since “infodemia” is also a serious public health problem^3^. At the same time, it is recognized that a strong combination of governance, regulation, community surveillance, social participation, and the intelligent use of big data and digital technologies has been decisive in combating the virus in countries such as China^4^. 

Faced with the need for definitions on guidelines and approaches for research in epidemic contexts, we start with three initial assumptions in regard to the epistemic background to arrive at the proposition of this essay. These assumptions of substantiation are taken collectively as a framework and theoretical justification: 

The pandemic is an inextricably cultural, political, and economic phenomenon and arouses narratives that try to give meaning, either by way of refusal or urgency, to inscribe it in a symbolic order^5^.

Presently, “the laboratory is everywhere” because of this privileged space of analysis of the scientific fact that “an extensive and heterogeneous network” is produced (i.e., not only the network of scientific communities that produces, stabilizes, and gives credibility to discoveries, but the network that brings to society the products of this laboratory). From the point of view of historiographical epistemology, laboratories connect “in the transnational arena of global science-they are in the world” and move the world. The utterances produced there (for the control of an “actor” virus) are spread in wide spaces, from hospitals, media and government offices^6^.

From the historiographical and constructivist epistemology of Ludwik Fleck, knowledge will always have a collective, cooperative, and interdisciplinary character within certain social and cultural conditions. Science is linked to communities of thought that share a style and body of practices. These collectives define “what is a problem,” “how to deal with the problem,” and “under what criteria to judge results,” thereby developing socialization processes that guarantee the relative stability of the group, its values, norms, and abilities. Despite this, as a source of innovation, different groups and fields interact with each other, translating into their style of thinking a scientific fact produced by another group, enriching it, and being modified by it. Thus, knowledge is produced in contingent and negotiable borders (zones of interaction-interdisciplinarity) and around border objects (weakly appropriated by common use, but strongly structured for specific uses)^7^.

By derivation of these assumptions, COVID-19 is affirmed as an object of knowledge that expresses the character and translational demands of (and regarding) science (utterance). This requires the assumption that in transactions for the appropriation of knowledge, the thought collectives sympathize and also dispute among themselves. Paradoxically, they position themselves in the insurmountable balance between the rigidity and flexibility of the style of thinking, between competing with uncertainty and seeking autonomy and prestige, and between defending borders (esoteric/private spheres) and not reducing themselves to closure and obsolescence^7^. 

From the previous statement, it is necessary to deal with what such needs of translation or critical appropriation indicate in terms of new guidelines and requirements for health research. Given the breadth of the field of health and a purposeful thematic choice, the focus on research pertaining to health work in the context of a pandemic is centralized. 

Therefore, the objective of this communication is to propose theoretical and methodological elements for research on health work in the context of a pandemic. This is a reflective essay, which supports the rationale for the research project on the topic of health work in the city of Manaus, state of Amazonas, Brazil. 

The severe acute respiratory syndrome coronavirus 2 (SARS-CoV-2) pandemic has brought to light ethical and legal devices that guide healthcare systems in their close relationship with the findings of the basic and applied sciences. More than that, it revealed the need to qualify the dialogue between “close but distant” areas. Qualified dialogue should mean minimal reciprocity regarding perspectives, needs, and tools relatively foreign to their thought community but which share epistemological objects, as in the case of COVID-19.

The application of normative and legal measures to contain COVID-19 and, ultimately, produce a solution has demanded adjusting health systems, as well as training and carefully analyzing resources and conditions aimed at expanding capacities and protecting the workforce. The preparation of health services and human resources is essential because of the association between transmission of the virus and inadequate protection, overwork, frustration, exhaustion, and illness among health workers^8^ ^,^ ^9^. The high demand for care and the contingent structure of services, in addition to inadequate working conditions, impact the quality of care and the physical and mental wear and tear of workers. 

When observing the actual work in different services that integrate healthcare in cases of COVID-19, issues unresolved by rules and protocols still need to be understood. Why, amid so much information, do workers develop other responses and arrangements, take risks, or make choices outside of protocols? How do work environments influence these choices? It is assumed that prescriptions define objectives and how to achieve them, but they are made by society and institutions as well as by individuals and groups of workers; they articulate the official and the unofficial and the continuous debate regarding norms^10^.

To support theoretical and methodological alternatives associated with future research agendas regarding health work, three guiding axes are proposed: 1) the construction of work environments as an analytical component, 2) the approach of ergology as a potential , and 3) the centrality of the experiences of the worker-subject. Before they represent a claim of completeness to the problems that involve the reality of the work, they point to an exemplary approach with the potential to foster mutual understanding between fields of knowledge.

For this approach, research questions oriented by axes for a research agenda are illustrated based on the findings of new problems and consequences for health work (e.g., infopandemia, protocols, standards, safety/risk issues, specific loads/conditions) (Figure 1).

**FIGURE 1:**
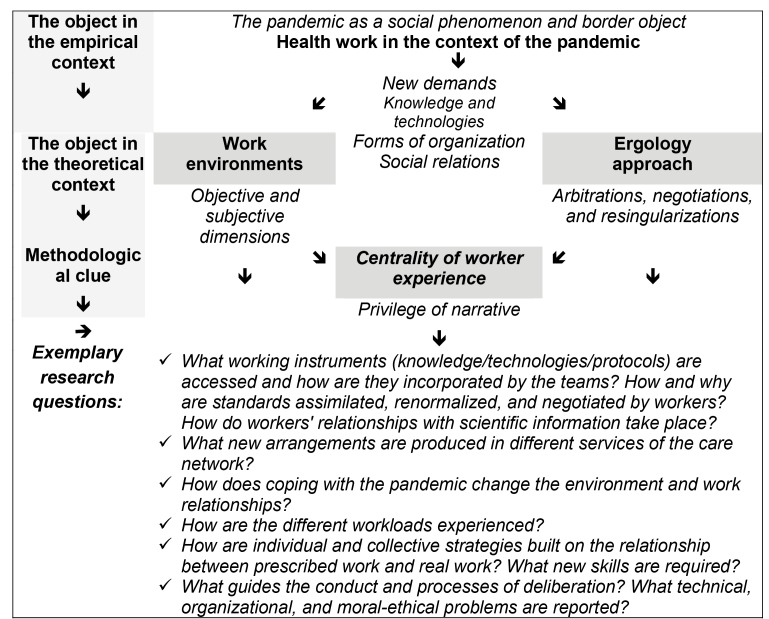
Exemplary configuration of a research agenda on health work in the context of a pandemic

The first axis/element refers to the constructs of healthy working environments in health services in pandemic scenarios. A healthy work environment is considered to be one in which workers and managers collaborate for continuous improvement in the protection and promotion of the safety, health, and well-being of all workers and sustainability of the work environment, taking into account some considerations established on the basis of previously determined needs^11^.

The relevance of a healthy work environment is reinforced by the exposure of health workers to the risks of illness or wear and tear related to the work environment. A relationship with the environment is not limited to the meaning of biosafety; rather, it incorporates subjective dimensions that involve safe work, vulnerabilities, working conditions and organization, psychosocial repercussions of risks and accidents, and relationships between prescribed work and actual work, among others. Thus, in a broader sense, the work environment includes both the physical and psychosocial environment of work. By highlighting the objective and subjective dimension, the second axis is reached, especially to the proposals of Ergology and contributions by the philosopher Yves Schwartz.

This second axis assumes work activity as a historical product, expressed in knowledge, technologies, forms of organization, procedures, and social relations present in productive systems. Antecedent norms, components of the prescribed work, are not absolute determinants of workplace reality. Every work activity will always be resingularization or partial renormalization-in other words, a use of oneself “by oneself” and “by others,” involving choices, arbitrations, and negotiations of this use of oneself. Hence, the concept of dramatics of the use of self emerges, giving light to this problematic negotiation of the uses of self in real work, lived in concrete situations^12^ ^,^ ^13^.

 The rules, routines, and protocols-so valued in a work environment that must link workers, training, and different experiences, as in the case of health teams-never eliminate the arbitrage of each worker in practices never fully standardized. To understand the collective dimension of work, we can use the notion of “relatively relevant collective entities” (RRCEs) in the cooperative processes that are not limited to predefined hierarchical definitions because they are built into everyday life, where information circulates in different directions and pathways under invisible and floating borders^13^ ^,^ ^14^ ^,^ ^15^. The RRCEs guide the work process according to references and logic specific to the activity. They are collective entities because they involve the search for several workers by effectiveness; they are relevant for understanding how work happens, and they are relative because the variable boundaries are formed from the acts of work related to people and the needs and histories of organizations^10^.

The centrality of real work experiences is the third axis/element to support the analysis of work. It is fundamental to analyze the ways in which workers manage individual and collective demands and efforts in spaces made complex by relationships involving interests, perspectives, and diverse work instruments. If health work alone requires an understanding of the centrality of the worker in the production of care, in critical scenarios, such as the COVID-19 pandemic, there are new and challenging elements to the operating intelligence relevant to the services, with new problems, threats, and capacity reconstruction. This can only be understood from what workers have to say about their ways of coping and reinventing in the face of the unforeseen and the negotiation of priorities and commitments. This axis articulates the first two and points to an important direction of the method-an appreciation for the narrative as a way of accessing experience and its individual-collective dynamics.

Through this reflective essay, we have presented some propositions of a research project based on the assumption of the relevance of the dialogue between a specific field of medical science and work studies. It is believed that the field of tropical medicine promotes knowledge that reflects the real work of those who incorporate, translate, and apply laboratory knowledge. There is considerable interest in approaching this area in terms of the potential contributions of the human and social sciences and the applicable methodologies.
